# Characterization and Discrimination of Volatile Compounds in Chilled Tan Mutton Meat during Storage Using HiSorb-TD-GC-MS and E-Nose

**DOI:** 10.3390/molecules28134993

**Published:** 2023-06-25

**Authors:** Ningxia Bu, Qi Yang, Juan Chen, Yongqin Li, Dunhua Liu

**Affiliations:** 1 College of Animal Science and Technology, Ningxia University, Yinchuan 750021, China; 18295082656@163.com; 2 Ningxia Veterinary Drugs and Fodder Inspection Institute, Yinchuan 750011, China; 3 College of Food Science and Engineering, Ningxia University, Yinchuan 750021, China

**Keywords:** chilled Tan mutton, HiSorb-TD-GC-MS, E-nose, volatile compounds, discrimination

## Abstract

Chilled Tan mutton is currently the mainstream of Tan mutton production and consumption in China, but the reports on chilled meat quality evaluation and shelf-life discrimination by volatiles are limited. This study aimed to investigate the changes of volatile compounds in chilled Tan mutton at four storage stages (1d, 3d, 5d, 7d) in order to differentiate the various storage stages. An analysis protocol was established for the characterization and discrimination of the volatiles in chilled Tan mutton based on high capacity sorptive extraction-thermal desorption-gas coupled with chromatography-mass spectrometry (HiSorb-TD-GC-MS), electronic nose (E-nose), and multivariate statistical analysis. A total of 96 volatile compounds were identified by HiSorb-TD-GC-MS, in which six compounds with relative odor activity value >1 were screened as the key characteristic volatiles in chilled Tan mutton. Four storage stages were discriminated by partial least squares discriminant analysis, and nine differential volatile compounds showed a variable importance for the projection score >1, including octanoic acid, methyl ester, decanoic acid, methyl ester, acetic acid, heptanoic acid, methyl ester, propanoic acid, 2-hydroxy-, methyl ester, (ñ)-, hexanoic acid, propanoic acid, butanoic acid, and nonanoic acid. With the volcano plot analysis, hexadecanoic acid, methyl ester, was the common volatile marker candidate to discriminate chilled stages of Tan mutton. Meanwhile, E-nose could discriminate chilled Tan mutton at different storage stages rapidly and efficiently using linear discriminant analysis. Furthermore, E-nose sensors could obtain comprehensive volatile profile information, especially in esters, acids, and alcohols, which could confirm the potential of E-nose for meat odor recognition. Thus, this analysis protocol could characterize and discriminate the volatiles in chilled Tan mutton during storage.

## 1. Introduction

Tan sheep, an excellent local ovine breed, are mainly distributed in the temperate zone or semi-arid temperate transitional areas in China, belong to the national second level protection breed, and are part of a national agricultural product geographical indication model [1,2]. Ningxia Hui Autonomous Region is the principal Tan sheep-producing area in China. The production continually increased from 2015 to 2021, with an average annual growth rate of 27.32% [3]. Chinese consumers prefer tan sheep due to their high protein, low levels of intramuscular fat and saturated fatty acids, delicious meat, and unique flavor [3,4]. At present, Tan mutton is mainly sold and circulated as carcass after slaughter, with a lack of produce that has been finely and deeply processed. Therefore, the quality maintenance of Tan mutton during circulation has become a research hotspot [5,6]. Chilled meat refers to the raw meat that has been rapidly cooled after slaughter by strict implementation of the quarantine system so that the carcass temperature drops to 0–4 °C within 24 h and remains at 0–4 °C during subsequent processing, circulation, and distribution [7]. Due to the advantages of maintaining meat quality, increasing palatability, and reducing nutritional loss, chilled meat has replaced fresh raw meat in the meat market [8]. However, the short shelf life of chilled meat is a major problem because of the existence of spoilage microorganisms [9].

Volatile flavor, one of the sensory qualities of meat, is important for evaluating raw meat quality and shelf life and will affect consumers’ purchasing behavior to a large extent [9,10]. During the maturation and storage of raw meat after slaughter, amino acids, reducing sugars, nucleotides, and other flavor precursor substances undergo a series of biochemical reactions to produce small molecules of volatile compounds and dictate the sensory properties and cooking quality of cooked meat products, and these reactions vary greatly with different storage conditions [1,11]. The control and regulation of volatile compounds in raw meat is a global research hotspot [12]. Currently, the research on the volatiles of Tan mutton focuses on the cooked meat [13,14]. However, there are few studies on volatile substance in raw Tan mutton to evaluate storage methods and shelf life.

Electronic nose (E-nose) is a volatile analysis system based on volatile components of samples which mimics the human olfactory system and uses gas sensors to establish response curves to quickly identify volatile composition [15]. The rapid, non-destructive, and real-time analysis can be used to describe the overall flavor profile of various samples, identify meat freshness, and detect adulteration, in combination with statistical analysis methods [16,17]. A high-capacity sorptive extraction (HiSorb) technique using polydimethylsiloxane (PDMS) adsorbent has the characteristics of large extraction quantity and wide applications [18]. Compared with traditional headspace (HS) and solid-phase microextraction (SPME), HiSorb has higher sensitivity, a larger capacity, and a lower detection limit, with the advantages of the low-temperature enrichment and rapid temperature rise analysis mode of thermal desorption (TD), and can provide the volatile substance information of the sample more comprehensively [19,20]. HiSorb-TD, as an emerging pre-treatment method for volatile substances, is usually combined with gas chromatography-mass spectrometry (GC-MS). At present, it has been applied in the detection of environmental air [21,22] and chemical material security [23,24], but not in food, especially in raw meat. 

Currently, to our knowledge, the characterization and discrimination of chilled Tan mutton during cold storage by flavoromics are rarely studied. Therefore, in this study, the composition and differences of volatile substances in chilled Tan mutton at four storage stages (1d, 3d, 5d, 7d) were analyzed and characterized using HiSorb-TD-GC-MS and E-nose technology. Moreover, principal component analysis (PCA), partial least squares discriminant analysis (PLS-DA), and linear discriminant analysis (LDA) were used to identify volatile substances and discriminate storage stages in chilled Tan mutton. Furthermore, the correlation between main volatiles and E-nose sensors were analyzed. This study may lay a theoretical foundation for the rapid identification and comprehensive quality control of chilled Tan mutton.

## 2. Results and Discussion

### 2.1. HiSorb-TD-GC-MS of Chilled Tan Mutton during Storage

#### 2.1.1. Volatile Profiles

**Figure 1:**
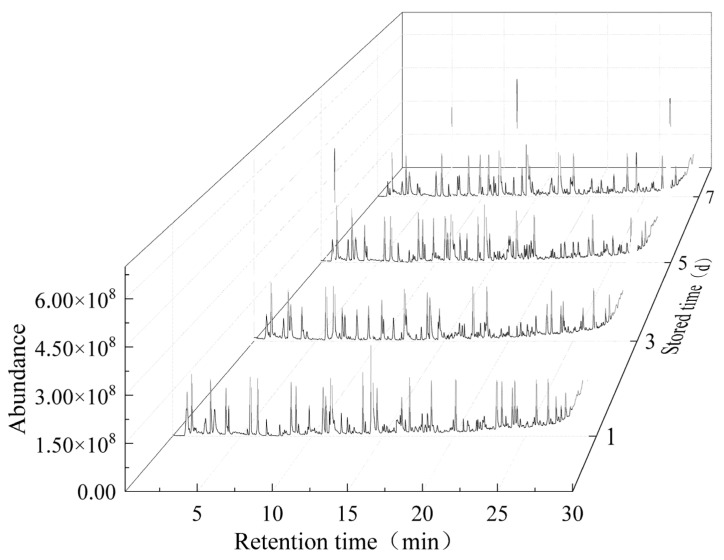
Total ion chromatogram of volatile compounds in chilled Tan mutton during storage.

In order to identify variations of volatile compounds in chilled Tan mutton meat at different storage stages, HiSorb-TD-GC-MS was applied to analyze volatile profiles. The peak signal distributions were similar in different samples. However, the peak signal intensities were different (Figure 1), indicating that the volatile profiles were similar, but the contents of volatile compounds were different at stored stages. As shown in Table 1, 96 volatile compounds were identified, including 23 esters, 18 alkanes, 18 alcohols, 16 acids, 10 aldehydes, 6 ketones, and five others. The total contents of volatile compounds at 1 d, 3 d, 5 d, and 7 d were 1391.76, 1852.30, 1513.26, and 3167.11 µg/kg, respectively. The content of volatile compounds in 7 d was significantly higher than those of the other periods, indicating that these compounds could be produced through chilled storage at 4 °C for 7 d. It has been reported that the flavor-related carbohydrates and free amino acids were accumulated under the action of the key enzymes involved in glycolysis and hydrolysis of structural proteins from day 4 to day 8 postmortem in Tan meat, which played crucial roles in improving meat volatile compounds [25].

**Figure 2:**
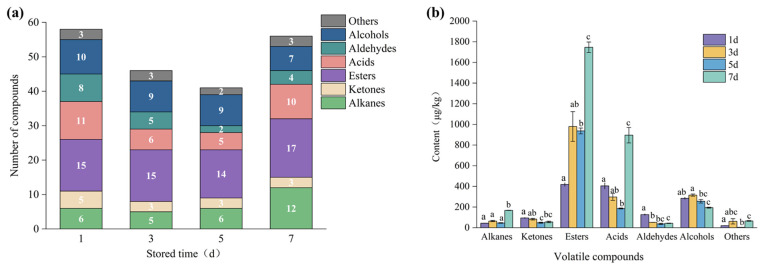
(**a**) The number of volatile compounds in chilled Tan mutton during storage; (**b**) The content of volatile compounds in chilled Tan mutton during storage. Different superscript letters represent statistically significant differences (*p* < 0.05).

As shown in Figure 2a, the number of volatile compounds firstly decreased and then increased during storage, with 58, 46, 41, and 56 in 1d, 3d, 5d, and 7d, respectively. The types and contents of esters, acids, and alcohols were higher than other compounds (Figure 2b), accounting for more than 70% of the total content. Meanwhile, the contents of esters and acids in 7 d were significantly higher than those in the first three storage periods (*p* < 0.05), probably due to the results of continuous esterification reactions of esterase and acidogenesis of exogenous and endogenous microbes [26].

Esters are the most abundant component in all the samples, with 30.05–61.92% of the total content. In general, esters are synthesized by esterification between alcohols and free fatty acids, which can produce a fruit aroma and a sweet taste with a low odor threshold [27]. Numerous studies have shown that esters existed widely in raw meat, such as lamb, rabbit, and beef [1,28,29]. The results showed that the contents of methyl caproate, methyl octanoate, methyl heptanoate, methyl decanoate, and other methyl esters were relatively high in the detected ester compounds, accounting for more than 80%, and tended to increase with the extension of storage period, which could be the results of continuous esterification of alcohols and acids at the late stages of storage. The high content of ester compounds provided a sweet taste for the flavor of chilled Tan sheep meat.

Short-chain fatty acids are regarded as the key contributors of distinctive “mutton flavor” [30]. Acetic acid, propanoic acid, hexanoic acid, and nonanoic acid were the main acids identified in this study, accounting for 56.37–85.56% in all the samples. As shown in Figure 2b, the content of acids in 7d was significantly higher than those in the first three storage periods (*p* < 0.05), which could be due to the fat oxidation and microbial metabolism [26], indicating that 7d of storage may be the critical period for the substantial increase of “mutton flavor” in chilled Tan mutton meat. 

Alcohol compounds provide flower, fruit, and rose flavor, which are generally considered to have a relatively higher odor threshold and contribute less to flavor, unless they exist in a highly concentrated or unsaturated form [31]. Alcohols were the second largest group of compounds detected, which first increased and then decreased during storage, with a significant decrease in 7d (*p* < 0.05). The reduction may be because the alcohols serve as the major substrate involved in esterification reaction of microorganisms [32]. As shown in Table 1, only two branched-chain alcohols, 1-octen-3-ol and 2-octen-1-ol, (E)-, were detected in all four stored periods. The two alcohols were reported to have a significant contribution to the aroma of mutton meat due to their lower odor threshold [12,33]. Meanwhile, the content of 1-octen-3-ol was the highest in alcohols, ranging from 56.69 to 89.73 µg/kg, which presented a strong green, mushroom, earthy, and oily smell [34]. It was produced by the linoleic acid under oxidation of lipids and is one of the main volatile alcohols in beef, duck meat products [35,36], and many aquatic products [37,38].

Aldehydes, commonly found in mutton meat with a relatively low odor threshold, were considered to be the representatives of volatile substances in ruminant’s meat [39,40]. In this study, ten kinds of aldehydes were indentified, and their contents tended to decrease with prolonged chilled storage time, which may be due to conversion into acids or alcohols [41]. Among them, the contents of hexanal, nonanal, 2-octenal, (E)-, 2-nonenal, (Z)-, decanal, and benzaldehyde accounted for more than 80%. Moreover, hexanal, an oxidation product of unsaturated omega-6 fatty acids, with a paint-like, apple taste, and leaf fragrance, was identified during the whole storage, indicating that it contributed to the flavor of mutton, which is consistent with the findings of Karabagias et al. [11]. However, aldehydes at high concentrations produce rancidity or other unpleasant odors because of lipid oxidation. Therefore, they have become the major indicators for the freshness and storability of meat [11,42]. 

Most ketones have an obvious milk or fruit flavor with a relatively high odor threshold [34]. Six ketones were found in this study, with the highest contents of 2(5H)-furanone, 3-methyl-and 2-pentadecanone. These ketones represented a minor fraction of volatile compounds in Tan meat. Finally, the content of alkanes in volatile compounds was less than 5.3% and reached a peak of 167.73 µg/kg in 7d (*p* < 0.05), but they had a minimal effect on the formation of the overall flavor of Tan mutton meat due to the higher odor threshold [43].

#### 2.1.2. ROVA Analysis

The volatile perception was determined by both the concentration of the compounds and the odor threshold value. The relative odor activity value (ROAV) is an index to estimate the contribution of each compound to the overall volatile profile by the relative concentration [44]. In general, volatiles with ROAV > 1 are commonly regarded as important contributors to key odors [45]. There were six key volatile compounds with ROAV > 1, including two aldehydes, one ketone, one ester, and one alcohol (Table 2). Among them, heptanoic acid, methyl ester, and 1-octen-3-ol were common key volatile compounds at all four storage stages and had a great impact on the overall odor of chilled Tan mutton. 1-octen-3-ol was the most abundant compound among the common key volatiles, and was reported as the potential marker in pre- and post-rigor roasted mutton [46].

#### 2.1.3. Discrimination of Volatile Compounds in Chilled Tan Mutton during Storage

**Figure 3:**
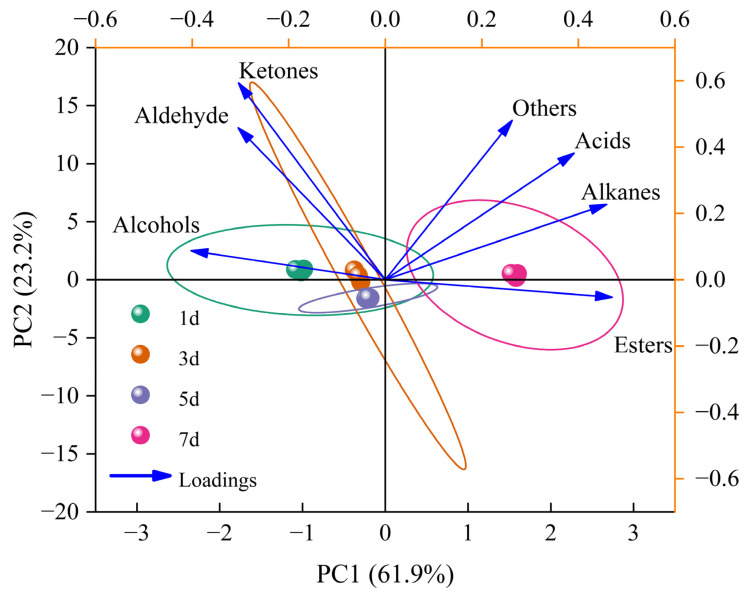
PCA of volatile compounds in chilled Tan mutton during storage.

To obtain more information of the volatiles and discriminate Tan mutton at different storage stages, principal component analysis (PCA), partial least squares discriminant analysis (PLS-DA), and volcano plot analysis were performed. PCA can realize data transformation of feature vector matrix and can transform correlation variables into linear uncorrelated variables with minimum variance information loss through dimensionality reduction, thus simplifying further analysis [48]. As shown in Figure 3, the accumulative variance contribution of the first two principal components were 61.9% and 23.2% (a total of 85.1%), suggesting that the two principal components covered almost all volatile information. Obviously, 3d and 5d were relatively close and far away from 1d and 7d in spatial regions, especially 7d, indicating that 3d and 5d had relatively similar volatile components and 7d was significantly different from the others. The differences in volatiles between 1d and 7d were mainly caused by PC1, and their projection positions were located on the negative and positive axes of the PC1 axis, respectively. These results demonstrated that four storage periods in chilled Tan mutton could be separated by the HiSorb-TD-GC-MS analysis of volatiles and divided into three groups (1d, 3d, and 5d, 7d) by PCA. In addition, the PCA loadings of alcohols, aldehydes, and ketones were associated with 1d, 3d, and 5d, while esters, alkanes, acids, and others were associated with 7d.

**Figure 4:**
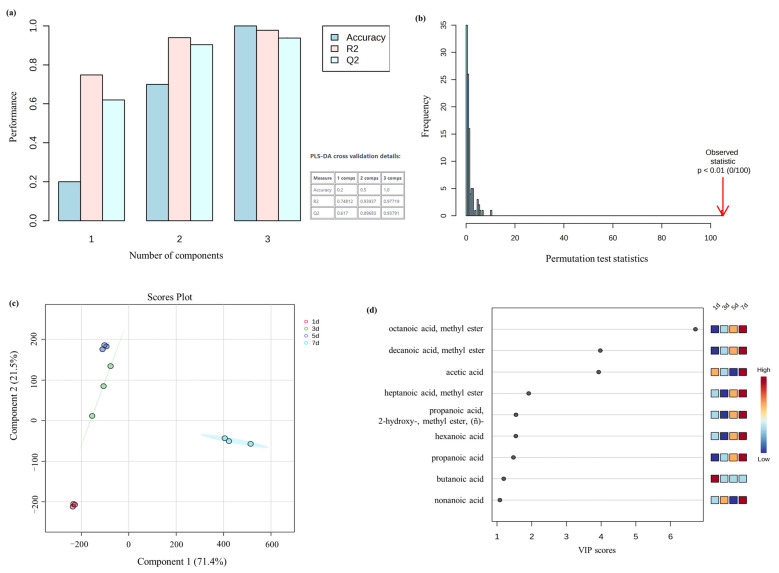
(**a**) Cross-validation of volatiles to estimate the predictive ability of the PLS-DA model; (**b**) The result of permutation test; (**c**) Score plot of PLS-DA; (**d**) The volatiles with VIP > 1.

PLS-DA is a supervised discriminant analysis method, which can establish a discriminant model to distinguish samples according to their volatiles [49]. The storage stages were selected as normalization factors for sample-specific normalization without data transformation and scaling. As shown in Figure 4a, R^2^ and Q^2^ were 0.977 and 0.938, respectively, indicating that the model had good predictability and repeatability with no overfitting [50]. Additionally, the result of a permutation test conducted 100 times indicated that the model was significant (*p* < 0.01, Figure 4b). Component 1 and component 2 account for 71.4% and 21.5% (a total of 92.9%) (Figure 4c). Tan mutton at four storage stages could be completely separated. Meanwhile, variable importance for the projection (VIP) of the PLS-DA model was calculated to identify differential volatile compounds. Generally, volatiles with VIP > 1 are considered to have a significant contribution to the odor [51]. As shown in Figure 4c, nine volatiles with VIP > 1 were screened, including octanoic acid, methyl ester (VIP = 6.72), decanoic acid, methyl ester (VIP = 3.98), acetic acid (VIP = 3.93), heptanoic acid, methyl ester (VIP = 1.91), propanoic acid, 2-hydroxy-, methyl ester, (ñ)- (VIP = 1.55), hexanoic acid (VIP = 1.54), propanoic acid (VIP = 1.47), butanoic acid (VIP = 1.19), and nonanoic acid(VIP = 1.08).

**Figure 5:**
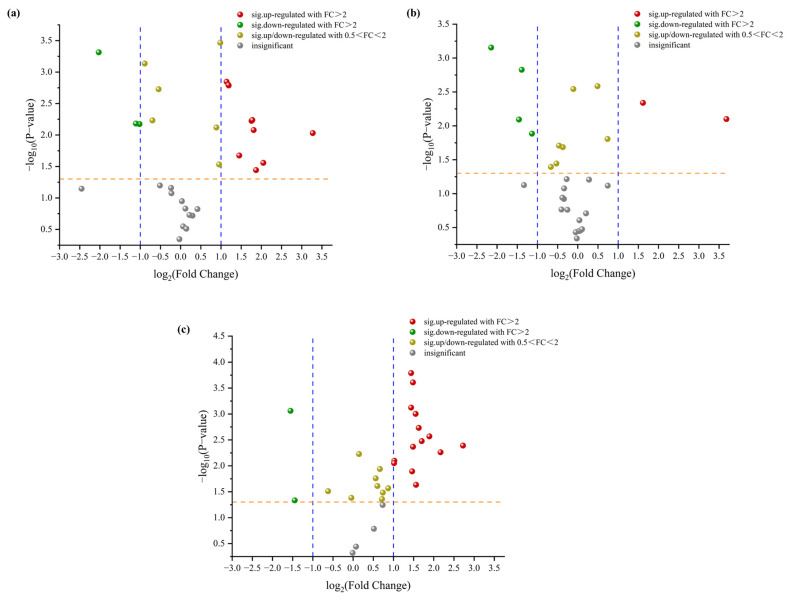
Volcano plot of volatile compounds in chilled Tan mutton during storage: (**a**) 3d vs. 1d; (**b**) 5d vs. 3d; (**c**) 7d vs. 5d. The dotted lines in yellow and blue represented *p* value = 0.05, fold change = 0.5 and 2, respectively.

Finally, in ordor to identify the potential volatile markers for different chilled stages of Tan mutton, volcano plot analysis between adjacent samples was performed (3d vs. 1d, 5d vs. 3d, and 7d vs. 5d) and is presented in Figure 5. The threshold was set to a fold change >2 and a t test *p* value < 0.05 to remove the less statistically significant compounds, the red spots represent an up-regulation, and the green spots represent a down-regulation. These up- or down-regulation spots were the volatile marker candidates that could differentiate the chilled storage stages of Tan mutton. As listed in Table 3, 13 volatiles were found to be the marker candidates between 3d and 1d (ten up-regulated, three down-regulated). Among them, esters and acids were the highest in up-regulated compounds, indicating that a large number of esters and acids were produced in the early stage of chilled storage. In the intermediate stage (5d vs. 3d), only two and four compounds were up-regulated and down-regulated, respectively, which may help explain the close proximity between 3d and 5d in the PCA result. Meanwhile, the contents of 1-octen-3-one, 1-hexadecanol, and 2-methyl- increased significantly, indicating that ketones and alcohols dominated the compound production at this stage. Immediately posterior, with the progress of storage period, complex and convoluted metabolic changes occurred in volatile compounds, resulting in a significant increase of volatiles in the late storage stages. Meanwhile, 17 volatiles were screened as the potential volatile markers between 7d and 5d (15 up-regulated, 2 down-regulated), which could be used for the identification of these two stages. Moreover, hexadecanoic acid and methyl ester, as the common volatile markers throughout the storage period, played an important role in the discrimination of chilled Tan mutton at different storage stages. 

### 2.2. E-Nose Analysis of Chilled Tan Mutton during Storage

#### 2.2.1. Radar Chart and PCA of E-Nose Sensors

**Figure 6:**
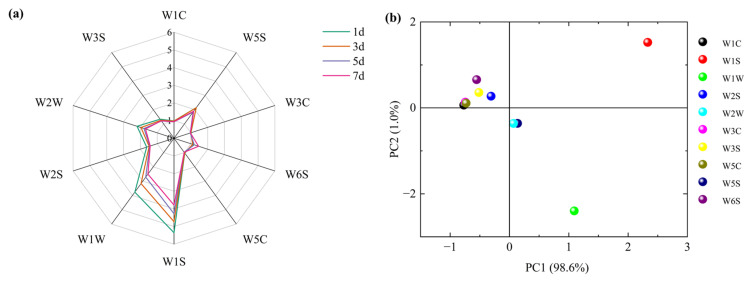
(**a**) Radar chart of E-nose sensors in chilled Tan mutton during storage; (**b**) PCA of E-nose sensors in chilled Tan mutton during storage.

E-nose, an odor analysis system based on volatile components of samples, uses gas sensors to establish response curves to quickly identify odor components [52]. Because the process is rapid, objective, non-destructive, and real-time, E-nose has been used to describe the overall flavor profile of livestock products [16]. In this study, 64–66 s on the sample response curves were selected for radar mapping and PCA of ten E-nose sensors. As shown in Figure 6a, the radar chart shapes on the ten sensors were similar, indicating that the flavor profiles of chilled Tan mutton at different storage stages were roughly the same. Meanwhile, the response values of W1S, W1W, W2W, W5S, and W6S sensors were higher than the others, with W1S (sensitive to methane and hydrocarbons) being the highest, followed by W1W (sensitive to many terpenes and sulfides). In addition, a significant reduction of response values occurred in these two sensors compared with the others, suggesting that methane, hydrocarbons, terpenes, and sulfides in Tan sheep meat may be gradually transformed into other substances during storage. However, this result was not the same as the result of HiSorb-TD-GC-MS. In addition, W2S, W3S, W5C, W3C, and W1C sensors had low response values and changes.

The contribution rate of E-nose sensors obtained through PCA are depicted in Figure 6b, which was conducive to identifying characteristic sensors used for distinguishing samples [27]. The accumulative variance contribution of the first two principal components was 99.6%, which covered almost all volatiles information. Obviously, the sensors with a large contribution to PC1 were W1W and W1S, and W1S had the largest contribution to PC2. Therefore, W1W and W1S sensors played a key role in distinguishing the chilled Tan mutton at different storage stages.

#### 2.2.2. PCA and LDA of E-Nose Response Value

In order to evaluate the identification potential of E-nose on chilled Tan mutton samples, the PCA and LDA of E-nose response values were performed using the WinMuster analysis system of the E-nose device.

**Figure 7:**
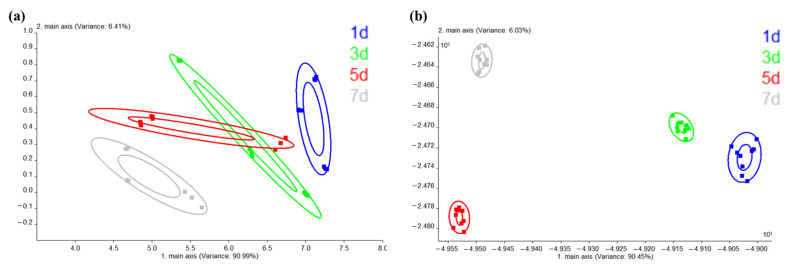
(**a**) PCA of E-nose response value in chilled Tan mutton during storage; (**b**) LDA of E-nose response value in chilled Tan mutton during storage.

As shown in Figure 7a, the total contribution rate of the first two principal components in PCA was 97.4%, which can represent the overall information of Tan mutton samples. These four samples were divided into three clusters (1d, 3d, and 5d, 7d) according to the E-nose response value, which was consistent with the PCA of volatile compounds. Data points from 3d and 5d of storage partially overlapped, indicating that there was little change in some volatile substances in Tan mutton meat at the two storage stages. Meanwhile, data points on 1d and 7d of storage had no crossover and a long distribution distance, suggesting that the flavor profiles at these two storage stages were significantly different. This may be because the protein, fat, and other substances in meat were degraded and volatile substances were produced by protein hydrolysis, lipid oxidation, and glycolysis, and the longer the storage period, the more significant the change [12]. In addition, the accumulative variance contribution of the first two principal components were 90.45% and 6.03% (a total of 96.48%, Figure 7b), indicating the effective discrimination functions of the LDA model [53]. Obviously, the data points of the four storage periods were not crossed and had a certain distance, suggesting that the four samples could be well separated by LDA of E-nose response value. Compared with PCA, LDA is a traditional pattern recognition and sample classification method with better separability, which can better show intra-group distribution and inter-group distance [54].

In summary, the E-nose analysis results showed that a combination of E-nose with LDA could distinguish the chilled Tan mutton at different storage stages, which had great potential in the intelligent, rapid recognition and preservation research of chilled Tan mutton meat.

### 2.3. Correlation between HiSorb-TD-GC-MS and E-Nose Results

**Figure 8:**
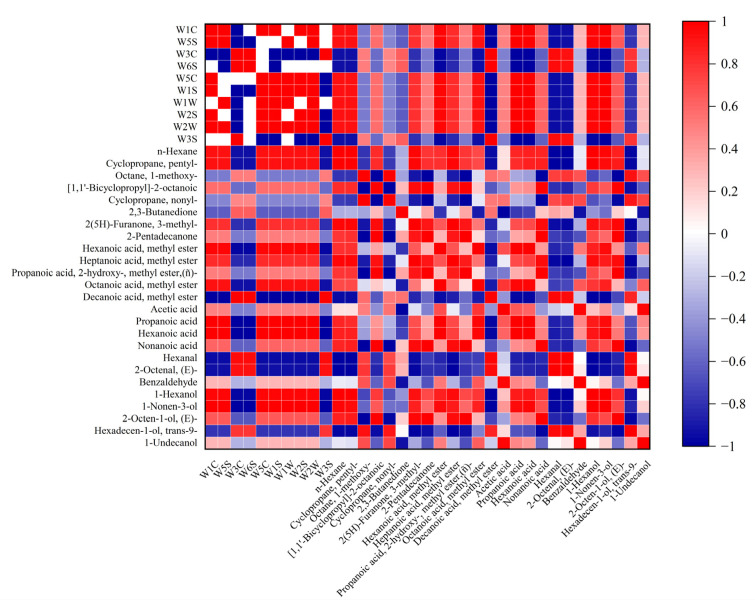
Correlation between main volatile compounds and E-nose sensors.

The E-nose system could successfully identify the chilled Tan mutton at different storage stages by the volatile profiles. Simultaneously, the contents of volatile compounds were analyzed by HiSorb-TD-GC-MS. A combination of these two techniques could achieve comprehensive evaluation and identification of volatiles in chilled Tan mutton. The volatiles with high contents were selected as the main volatile components to perform the correlation analysis with E-nose sensors. As shown in Figure 8, ten sensors could be roughly separated into two categories: W1C, W5S, W5C, W1S, W1W, W2S, W2W, W3C, W6S, and W3S. Among them, W1C, W5S, W5C, W1S, W1W, W2S, and W2W were positively correlated with n-hexane, cyclopropane, pentyl-, hexanoic acid, methyl ester, heptanoic acid, methyl ester, propanoic acid, hexanoic acid, 1-hexanol, and 1-octen-3-ol. Esters, acids, and alcohols were the main volatile components in chilled Tan mutton, which would explain their high correlation with the most E-nose sensors. Conversely, W3C, W6S, and W3S were found to be positively correlated with aldehydes (hexanal, 2-octenal, (E)-), ketone (2,3-butanedione), and other volatiles (decanoic acid, methyl ester, hexadecen-1-ol, trans-9-). Interestingly, they were located in the second quadrant with the short distance distribution in PCA plot of E-nose sensors (Figure 6b), indicating a certain similarity in their sensitivity to volatile substances. These results suggest that E-nose can obtain comprehensive contour information through the sensitivity of ten sensors to different volatiles so as to realize the identification of chilled Tan mutton at different storage stages.

## 3. Materials and Methods

### 3.1. Materials

2-methyl-3-heptanone (HPLC grade) was purchased from Dr Ehrenstorfer (Augsburg, Germany), and C_7_-C_40_ n-alkanes were purchased from Sigma-Aldrich Chemicals (St. Louis, MO, USA).

Six male Tan sheep (6 months old) were obtained from a pasture (Ning Xia Xin Hai Co., Yanchi, China) and slaughtered by a commercial slaughtering method. Twelve hind legs of Tan sheep (carcass weight 25 ± 5 kg) were excised from each carcass 30 min after exsanguination, and were then assigned into four groups (1d, 3d, 5d, 7d) randomly. Referring to the method in our previous study [55], all the samples were immediately transported to the laboratory under ice bag preservation and any visible external fat and connective tissues were trimmed, cut into pieces (average weight 150 ± 2.5 g), placed on plastic trays with transparent plastic covering, and stored at 4 °C until analysis. 

### 3.2. HiSorb-TD-GC-MS

Meat samples were homogenized using an automatic intelligence homogenizer (HM7300, Laipu Ltd., Beijing, China) at different storage stages. Following that, 5.0 g of the sample was weighed and put into a 10 mL headspace sample vial with an absorptive stick (PDMS, 5 cm, 65 μL, Markes, UK) and then heated by an HiSorb agitator (U-HSAG-20, Markes, UK) at 65 °C, 300 rpm, for 40 min. Following that, the absorptive stick was put into the sample tube and sequentially processed in the automatic thermal desorption system (TD100-xr, Markes, UK). The thermal desorption of the sample was carried out at 250 °C for 10 min using helium as a carrier gas at a flow rate of 50 mL/min. The volatile compounds were cryo-focused on the cold trap. The cold trap was then heated rapidly from 10 °C to 300 °C. In this manner, the volatile compounds desorbed from the sampling tube were transferred to the GC system. The whole system was maintained at 300 °C for 3 min in preparation for the next sample analysis.

Volatile compounds analysis was performed on a GC-MS system (Trace ISQ 150701, Thermo Fisher Scientific Ltd., Waltham, MA, USA) according to the method of Bai et al. [33], with some modifications. A TR-Wax capillary column [30 m (length) × 0.25 mm (internal diameter) × 0.25 μm (film thickness), Thermo Fisher Scientific Ltd., Waltham, MA, USA, was used for the separation of the volatile compounds. The GC oven temperature was programmed to start from 40 °C, held for 3 min, and then heated to 200 °C at a rate of 5 °C /min with a hold of 1 min, then heated to 230 °C at a rate of 10 °C /min with a final hold of 3 min. Helium was used as the carrier gas with a constant flow rate of 1 mL/min. The temperatures of the injector and ion source were 240 °C and 260 °C, respectively. Mass spectrometry was performed in electron collision mode (EI) with a voltage of 70 eV and a full scan mode of 40–600 amu.

Volatile compounds were identified through a mass spectral library (NIST 2.2), with a match of at least 750, and a series of n-alkanes (C_7_–C_40_) were used to determine the retention indices (RI) of each compound and were compared to the NIST database (https://webbook.nist.gov, accessed on 21 June 2023). Semi-quantitative analysis of the volatile compounds was carried out using 100 microliters of 2-methyl-3-heptanone (100 μg/mL, dissolved in methyl alcohol) as an internal standard according to the peak area ratio and the concentration of 2-methyl-3-heptanone [12].

### 3.3. E-Nose Analysis

The volatile profiles were characterized by an electronic nose (PEN3 Portable Electronic Nose, Airsense Analytics GmbH, Schwerin, Germany), which contains ten different metal oxide semiconductor (MOS) sensors to provide selectivity towards varied volatile compound classes (Table 4) [27]. 

For the analysis, 5.0 g of meat samples were put into a 20 mL headspace sample vial and equilibrated by incubation at 65 °C for 40 min. Next, the sample was sealed and left at room temperature for 10 min for testing. The self-cleaning time, automatic zeroing time, sample preparation time, and detection time of E-nose sensors were 80 s, 5 s, 5 s, and 100 s, respectively. The airflow rate was 400 mL/min. The sensor signal values of 64–66 s were selected for data analysis when a steady status could be maintained.

### 3.4. Statistical Analysis

Results were expressed as mean ± standard deviation (SD). Data were analysed by ANOVA, followed by Duncan’s test to analyze the significance of volatile compounds (*p* < 0.05) by SPSS software v19.0 (SPSS, Inc., Chicago, IL, USA). The relative odor activity value (ROAV) was calculated as the ratio of the odor activity value of each compound to the highest odor activity value [44]. The PCA and LDA of E-nose sensors were performed using the WinMuster analysis system of the E-nose device. PLS-DA model was established by MetaboAnalyst 5.0 (https://www.metaboanalyst.ca/MetaboAnalyst/, accessed on 21 June 2023) website. Multivariate statistical analysis and graphical work were performed by OriginPro software v2023 (OriginLab, Northampton, MA, USA). All experiments were repeated in triplicate.

## 4. Conclusions

In this study, the volatile components in chilled Tan mutton meat at four chilled storage stages (1 d, 3 d, 5 d, and 7 d) were characterized and discriminated by HiSorb-TD-GC-MS and E-nose. The multivariate statistical analysis showed that the total contents of 96 volatile compounds detected by HiSorb-TD-GC-MS tended to increase during storage, including 23 esters, 18 alkanes, 18 alcohols, 16 acids, 10 aldehydes, six ketones, and five others, and six volatile compounds with ROAV *>* 1 (1-octen-3-one, heptanoic acid, methyl ester, 2-nonenal, (E)-, decanal, 2,4-decadienal, and 1-octen-3-ol) were confirmed to be the key volatiles in the chilled Tan mutton. Notably, chilled Tan mutton at four storage stages could be discriminated by PLS-DA of volatile compounds, and 9 differential volatile compounds were identified using VIP method, including octanoic acid, methyl ester, decanoic acid, methyl ester, acetic acid, heptanoic acid, methyl ester, propanoic acid, 2-hydroxy-, methyl ester, (ñ)-, hexanoic acid, propanoic acid, butanoic acid, and nonanoic acid. Furthermore, volcano plot analysis identified 13, 6, and 17 volatiles marker candidates in 3d vs. 1d, 5d vs. 3d, and 7d vs. 5d, respectively. Among them, hexadecanoic acid and methyl ester were the common marker candidates to achieve the discrimination of different stages. Moreover, a combination of E-nose and LDA could not only depict the flavor profile of chilled Tan mutton but also rapidly identify the storage stages. Among them, W1W and W1S were the characteristic sensors, and the esters, acids, and alcohols with the highest concentrations in chilled Tan mutton were highly correlated with most E-nose sensors, which proved the rationality of E-nose in the identification of Tan mutton. Overall, the multivariate statistical analysis results of HiSorb-TD-GC-MS were highly consistent with E-nose, and a combination of the two methods could comprehensively analyze the difference of volatiles in chilled Tan mutton and rapidly identify storage stages. The results provide a theoretical basis for the quality control and maturation mechanism of Tan mutton in chilled storage.

## Figures and Tables

**Table 1:** GC-MS analysis of volatile compounds of chilled Tan mutton during storage.

RI	Volatile Compounds	CAS	Content (µg/kg)			
			1d	3d	5d	7d
Alkanes						
753	n-Hexane	110-54-3	-	41.26 ± 5.86 ^a^	18.79 ± 0.56 ^b^	18.23 ± 0.69 ^b^
644	Hexane, 2,4-dimethyl-	589-43-5	-	-	-	12.68 ± 1.23
743	Octane	111-65-9	1.92 ± 0.07 ^a^	7.01 ± 2.45 ^b^	2.78 ± 0.80 ^a^	7.67 ± 0.58 ^b^
832	2-Octene	111-67-1	0.92 ± 0.16	-	-	-
1096	Undecane	1120-21-4	18.01 ± 1.39 ^a^	8.89 ± 2.32 ^b^	9.07 ± 1.61 ^b^	13.76 ± 1.04 ^c^
1202	Tridecane	629-50-5	-	-	3.33 ± 0.23	-
1277	Tridecane, 3-methylene-	19780-34-8	-	-	-	3.20 ± 0.31
1304	Dodecane	112-40-3	-	1.92 ± 0.06	-	-
1447	Cyclopropane, pentyl-	2511-91-3	20.95 ± 0.70 ^a^	-	-	17.41 ± 0.62 ^b^
1450	Octane, 1-methoxy-	929-56-6	-	-	-	27.40 ± 0.93
1481	3-Tetradecene, (Z)-	41446-67-7	0.20 ± 0.01	-	-	-
1490	2-Undecenal	2463-77-6	1.27 ± 0.12	-	-	-
1497	Pentadecane	629-62-9	-	-	-	11.42 ± 0.69
1497	Hexadecane	544-76-3	-	5.20 ± 0.11 ^a^	4.82 ± 0.84 ^a^	5.33 ± 0.56 ^a^
1497	Pentadecane, 7-methyl-	6165-40-8	-	-	8.46 ± 0.77	-
1575	8-Heptadecene	2579-04-06	-	-	-	14.47 ± 1.04
1695	[1,1′-Bicyclopropyl]-2-octanoic	56687-68-4	-	-	-	14.69 ± 1.88
1815	Cyclopropane, nonyl-	74663-85-7	-	-	-	21.47 ± 0.99
Ketones						
1006	2,3-Butanedione	431-03-8	-	-	-	3.71 ± 0.64
1271	1-Octen-3-one	4312-99-6	6.71 ± 0.48 ^a^	4.70 ± 0.99 ^a^	14.39 ± 0.86 ^b^	-
1427	Acetoin	513-86-0	4.00 ± 0.19	-	-	-
1429	2-Propanone, 1-hydroxy-	116-09-6	13.27 ± 0.34	-	-	-
1500	2(5H)-Furanone, 3-methyl-	22122-36-7	65.83 ± 1.00 ^a^	68.65 ± 6.91 ^a^	26.21 ± 2.97 ^b^	43.08 ± 6.52 ^ab^
1858	2-Pentadecanone	2345-28-0	4.63 ± 1.69 ^a^	8.96 ± 1.20 ^ab^	7.49 ± 2.70 ^ac^	12.46 ± 1.03 ^bc^
Esters						
1017	Butanoic acid, methyl ester	623-42-7	5.75 ± 0.75 ^a^	15.73 ± 3.12 ^bc^	15.16 ± 0.53 ^ac^	56.22 ± 5.03 ^d^
1092	Methyl valerate	624-24-8	2.17 ± 0.51 ^a^	4.01 ± 0.69 ^a^	1.46 ± 0.13 ^a^	-
1152	Hexanoic acid, methyl ester	106-70-7	94.31 ± 13.55 ^a^	330.71 ± 67.01 ^ab^	381.13 ± 13.48 ^b^	129.86 ± 4.97 ^a^
1197	Octanoic acid, 4-methyl-, methyl ester	15870-07-2	-	14.71 ± 2.38 ^a^	15.07 ± 0.92 ^a^	44.45 ± 2.47 ^b^
1229	Heptanoic acid, methyl ester	106-73-0	36.69 ± 1.12 ^a^	35.92 ± 8.85 ^a^	59.84 ± 6.26 ^a^	161.99 ± 5.39 ^b^
1307	Hexano-dibutyrin	65235-12-3	1.59 ± 0.17	-	-	-
1312	n-Caproic acid vinyl ester	3050-69-9	18.05 ± 0.74 ^a^	-	28.55 ± 4.43 ^b^	-
1316	Allyl 2-ethyl butyrate	7493-69-8	-	-	-	21.32 ± 1.18
1317	Methyl 6-methyl heptanoate	2519-37-1	-	-	5.53 ± 0.97 ^a^	7.90 ± 2.48 ^b^
1343	Propanoic acid, 2-hydroxy-, methyl ester, (ñ)-	2155-30-8	55.64 ± 2.95 ^a^	47.18 ± 8.53 ^a^	57.23 ± 2.98 ^a^	159.97 ± 3.31 ^b^
1343	Octanoic acid, methyl ester	111-11-5	10.50 ± 7.91 ^a^	101.57 ± 14.78 ^b^	106.28 ± 3.80 ^bc^	477.21 ± 64.53 ^c^
1357	Formic acid, hexyl ester	629-33-4	10.97 ± 0.72	-	-	-
1418	Formic acid, heptyl ester	112-23-2	11.83 ± 0.52 ^a^	2.90 ± 0.12 ^b^	-	-
1427	Nonanoic acid, methyl ester	1731-84-6	-	38.57 ± 2.13 ^a^	-	80.03 ± 25.43 ^b^
1434	Decanoic acid, methyl ester	110-42-9	51.50 ± 0.61 ^a^	174.01 ± 23.62 ^a^	178.96 ± 33.83 ^a^	327.05 ± 28.70 ^b^
1455	Methyl 11-oxo-9-undecenoate	53613-55-1	4.15 ± 0.07	-	-	-
1479	4-Decenoic acid, methyl ester	1191-02-2	-	8.16 ± 0.44 ^a^	6.31 ± 0.52 ^a^	18.47 ± 1.03 ^b^
1494	Undecanoic acid, methyl ester	1731-86-8	-	-	-	8.79 ± 0.61
1552	Dodecanoic acid, methyl ester	111-82-0	15.56 ± 1.52 ^a^	19.06 ± 4.28 ^a^	15.03 ± 0.56 ^a^	48.83 ± 4.38 ^b^
1619	Benzeneacetic acid, methyl ester	101-41-7	-	4.93 ± 0.80 ^a^	-	3.54 ± 0.33 ^a^
1653	Butyl lactate	138-22-7	-	-	-	6.06 ± 0.70
1850	Methyl tetradecanoate	124-10-7	40.21 ± 0.84 ^ab^	46.77 ± 10.81 ^a^	35.87 ± 0.55 ^b^	100.43 ± 10.27 ^c^
2014	Hexadecanoic acid, methyl ester	112-39-0	59.23 ± 4.59 ^a^	135.23 ± 6.44 ^b^	30.54 ± 0.83 ^c^	94.31 ± 7.58 ^d^
Acids						
1417	Acetic acid	64-19-7	165.94 ± 13.29 ^a^	89.53 ± 17.55 ^b^	67.66 ± 13.34 ^b^	447.04 ± 57.48 ^ac^
1461	Formic acid	64-18-6	25.18 ± 5.65 ^b^	4.59 ± 0.78 ^a^	7.67 ± 2.86 ^a^	2.81 ± 0.18 ^a^
1466	Propanoic acid	79-09-4	12.60 ± 0.44 ^a^	52.14 ± 17.15 ^ab^	56.10 ± 3.35 ^b^	113.91 ± 15.42 ^b^
1485	Butanoic acid, 4-hydroxy-	591-81-1	4.80 ± 0.21	-	-	-
1487	Butanoic acid	107-92-6	103.25 ± 2.43	-	-	-
1490	Butanoic acid, 3-methyl-	503-74-2	-	-	-	24.97 ± 3.43
1491	2-Propenoic acid	79-10-7	3.51 ± 0.38	-	-	-
1490	Undecanoic acid, 2-methyl-	24323-25-9	-	-	-	7.04 ± 0.81
1626	Pentanoic acid	109-52-4	5.01 ± 0.07	-	-	-
1648	Crotonic acid	3724-65-0	-	-	-	5.57 ± 3.84
1658	2-Butenoic acid, (E)-	107-93-7	4.50 ± 0.07	-	-	-
1675	Cyclopropanetetradecanoic	52355-42-7	-	-	-	4.03 ± 0.24
1709	Hexanoic acid	142-62-1	30.16 ± 2.27 ^a^	-	45.84 ± 10.32 ^a^	124.15 ± 8.57 ^b^
1808	Heptanoic acid	111-14-8	4.73 ± 0.19 ^a^	10.42 ± 0.35 ^b^	8.60 ± 0.86 ^ab^	14.33 ± 1.92 ^b^
1901	Octanoic acid	124-07-2	-	38.36 ± 3.45	-	-
1993	Nonanoic acid	112-05-0	45.04 ± 0.64 ^a^	102.39 ± 6.03 ^b^	-	151.57 ± 16.59 ^b^
Aldehyde						
1087	Hexanal	66-25-1	38.82 ± 2.83 ^ab^	42.14 ± 1.31 ^b^	29.19 ± 5.09 ^a^	30.72 ± 1.50 ^ab^
1148	Heptanal	111-71-7	0.39 ± 0.16 ^a^	0.43 ± 0.13 ^a^	-	-
1317	2-Heptenal, (Z)-	57266-86-1	-	2.50 ± 0.37	-	-
1346	Nonanal	124-19-6	29.04 ± 1.74	-	-	-
1369	Undecanal	112-44-7	3.66 ± 0.30	-	-	-
1397	2-Octenal, (E)-	2548-87-0	-	-	7.86 ± 0.72 ^a^	5.11 ± 0.59 ^b^
1421	2-Nonenal, (E)-	18829-56-6	6.26 ± 0.75 ^a^	3.86 ± 0.90 ^b^	-	-
1422	Decanal	112-31-2	37.32 ± 0.78 ^a^	-	-	1.32 ± 0.31 ^b^
1433	Benzaldehyde	100-52-7	5.72 ± 0.74 ^a^	2.65 ± 0.63 ^b^	-	5.36 ± 0.35 ^a^
1618	2,4-Decadienal	2363-88-4	5.84 ± 0.19	-	-	-
Alcohols						
1205	1-Pentanol	71-41-0	33.89 ± 1.96 ^a^	45.25 ± 13.78 ^a^	32.78 ± 9.60 ^a^	-
1354	1-Hexanol	111-27-3	-	-	-	30.30 ± 1.64
1393	trans-2-Undecen-1-ol	75039-84-8	-	1.58 ± 0.19	-	-
1412	1-Hexadecanol, 2-methyl-	2490-48-4	-	0.91 ± 0.16 ^a^	11.68 ± 0.32 ^b^	-
1412	1-Octen-3-ol	3391-86-4	70.06 ± 1.78 ^a^	71.62 ± 0.67 ^ab^	56.69 ± 11.95 ^ab^	89.73 ± 3.86 ^b^
1416	1-Heptanol	111-70-6	8.06 ± 0.23 ^a^	-	5.70 ± 0.68 ^b^	-
1424	1-Hexanol, 2-ethyl-	104-76-7	10.79 ± 0.66	-	-	-
1429	2-Decen-1-ol	22104-80-9	-	2.02 ± 1.76	-	-
1465	2-Octen-1-ol, (E)-	18409-17-1	21.06 ± 0.30 ^a^	14.42 ± 0.52 ^ab^	14.19 ± 2.79 ^ab^	14.06 ± 1.59 ^b^
1473	2,3-Butanediol	513-85-9	24.21 ± 0.70 ^a^	-	-	0.60 ± 0.05 ^b^
1475	4-Methyl-5-decanol	213547-15-0	10.93 ± 0.51	-	-	-
1583	1-Hexadecanol, 2-methyl-	2490-48-4	-	-	0.34 ± 0.05	-
1612	Hexadecen-1-ol, trans-9-	64437-47-4	-	-	23.53 ± 2.19 ^a^	34.68 ± 3.56 ^b^
1729	Benzyl alcohol	100-51-6	-	-	-	4.45 ± 0.63
1747	1-Propanol, 2,2′-oxybis-	108-61-2	27.61 ± 2.90 ^a^	94.68 ± 14.56 ^b^	-	-
1753	1-Propanol,2-(2-hydroxypropoxy)-	106-62-7	38.69 ± 0.69 ^a^	76.47 ± 2.10 ^b^	107.32 ± 5.88 ^c^	-
1794	1-Undecanol	112-42-5	38.87 ± 2.00 ^a^	-	10.07 ± 1.94 ^b^	20.35 ± 1.70 ^c^
1880	2-butyl-1-Octanol	3913-02-8	-	13.44 ± 2.34	-	-
Others						
1031	Methylamine, N, N-dimethyl-	75-50-3	5.19 ± 0.97 ^a^	-	1.38 ± 0.09 ^b^	27.73 ± 2.79 ^c^
1184	Furan, 2-pentyl-	3777-69-3	-	3.33 ± 0.60 ^a^	2.10 ± 0.32 ^b^	-
1637	Acetamide	60-35-5	8.83 ± 0.32 ^a^	-	-	25.53 ± 1.12 ^b^
1762	Dimethyl sulfone	67-71-0	5.63 ± 0.38 ^a^	4.81 ± 1.05 ^a^	-	11.93 ± 0.38 ^b^
1714	Ethyl dodecyl ether	7289-37-4	-	53.79 ± 24.05	-	-

RI: retention indices. SD: standard deviation (n = 3). CAS: unique identification number of volatile compounds by comparison of mass spectra to NIST 2.2 library. “-”: no detected; Different superscript letters represent statistically significant differences (*p* < 0.05).

**Table 2:** Volatile compounds with ROAV ≥ 1 of chilled Tan mutton during storage.

NO.	Volatile Compounds	Threshold (μg/kg)	Odour Description	ROAV			
				1d	3d	5d	7d
1	1-octen-3-one	0.005	earthy, mushroom	100 ± 5.9	100 ± 17.17	100 ± 4.89	-
2	heptanoic acid, methyl ester	4	pineapple, fruity	0.68 ± 0.02	0.95 ± 0.19	0.52 ± 0.04	45.13 ± 1.23
3	2-nonenal, (E)-	0.08	fat	5.84 ± 0.57	5.12 ± 0.97	-	-
4	decanal	0.15	soap, orange peel, tallow	18.55 ± 0.32	-	-	9.8 ± 1.88
5	2,4-decadienal	0.07	seaweed	6.22 ± 0.16	-	-	-
6	1-octen-3-ol	1	mushroom, rose	5.22 ± 0.11	7.61 ± 0.06	1.97 ± 0.34	100 ± 3.52

ROVA: relative odor activity value. Thresholds and odour description were acquired from the literature [27,47], and the online database (http://www.odour.org.uk, accessed on 21 June 2023). “-”: no detected.

**Table 3:** Volatile marker candidates, fold change, and their regulations in chilled Tan mutton during storage.

3d vs. 1d			5d vs. 3d			7d vs. 5d		
Volatile Marker Candidates	Fold Change	Regulation	Volatile Marker Candidates	Fold Change	Regulation	Volatile Marker Candidates	Fold Change	Regulation
octane	3.65	up	1-octen-3-one	3.06	up	octane	2.76	up
butanoic acid, methyl ester	2.74	up	1-hexadecanol, 2-methyl-	12.84	up	butanoic acid, methyl ester	3.71	up
hexanoic acid, methyl ester	3.51	up	n-hexane	0.46	down	octanoic acid, 4-methyl-, methyl ester	2.95	up
octanoic acid, methyl ester	9.67	up	2(5H)-furanone, 3-methyl-	0.38	down	heptanoic acid, methyl ester	2.71	up
decanoic acid, methyl ester	3.38	up	methyl valerate	0.36	down	propanoic acid, 2-hydroxy-, methyl ester,(ñ)-	2.80	up
hexadecanoic acid, methyl ester	2.28	up	hexadecanoic acid, methyl ester	0.23	down	octanoic acid, methyl ester	4.49	up
propanoic acid	4.14	up				4-decenoic acid, methyl ester	2.93	up
heptanoic acid	2.20	up				dodecanoic acid, methyl ester	3.25	up
nonanoic acid	2.27	up				methyl tetradecanoate	2.80	up
1-propanol, 2,2′-oxybis-	3.43	up				hexadecanoic acid, methyl ester	3.09	up
undecane	0.49	down				acetic acid	6.61	up
pormic acid, heptyl ester	0.25	down				propanoic acid	2.03	up
benzaldehyde	0.46	down				hexanoic acid	2.71	up
						1-Undecanol	2.02	up
						methylamine, N,N-dimethyl-	20.09	up
						hexanoic acid, methyl ester	0.34	down
						Formic acid	0.37	down

**Table 4:** Sensor properties of the E-nose sensor.

Sensor Number	Sensor Name	Descriptions
1	W1C	Sensitive to aromatic, benzene
2	W5S	Sensitive to nitrogen oxides
3	W3C	Sensitive to ammonia and aromatic compounds
4	W6S	Sensitive to hydrogen
5	W5C	Sensitive to alkanes, aromatic compounds, and fewer polar compounds
6	W1S	Sensitive to methane and hydrocarbons
7	W1W	Sensitive to many terpenes and sulfide compounds
8	W2S	Sensitive to alcohols, aldehydes, and ketones
9	W2W	Sensitive to organic sulfides, aromatic compounds
10	W3S	Sensitive to long-chain alkanes

## Data Availability

The data is contained within the article.

## References

[1] Wang F., Gao Y., Wang H., Xi B., He X., Yang X., Li W. (2021). Analysis of volatile compounds and flavor fingerprint in Jingyuan lamb of different ages using gas chromatography–ion mobility spectrometry (GC–IMS). Meat Sci..

[2] Jia W., Li R., Wu X., Liu S., Shi L. (2021). UHPLC-Q-Orbitrap HRMS-based quantitative lipidomics reveals the chemical changes of phospholipids during thermal processing methods of Tan sheep meat. Food Chem..

[3] Li J., Tang C., Yang Y., Hu Y., Zhao Q., Ma Q., Yue X., Li F., Zhang J. (2023). Characterization of meat quality traits, fatty acids and volatile compounds in Hu and Tan sheep. Front. Nutr..

[4] Yang Y., Li J., Jia X., Zhao Q., Ma Q., Yu Y., Tang C., Zhang J. (2022). Characterization of the Flavor Precursors and Flavor Fingerprints in Grazing Lambs by Foodomics. Foods.

[5] Li D., Zhang H., Ma L., Tao Y., Liu J., Liu D. (2020). Effects of ficin, high pressure and their combination on quality attributes of post-rigor tan mutton. LWT.

[6] Fan N., Liu G., Zhang C., Zhang J., Yu J., Sun Y. (2022). Predictability of carcass traits in live Tan sheep by real-time ultrasound technology with least-squares support vector machines. Anim. Sci. J..

[7] Yuan L., Feng W., Zhang Z., Peng Y., Xiao Y., Chen J. (2020). Effect of potato starch-based antibacterial composite films with thyme oil microemulsion or microcapsule on shelf life of chilled meat. LWT.

[8] Huang X., Sun W., Li Z., Shi J., Zhang N., Zhang Y., Zhai X., Hu X., Zou X. (2022). Hydrogen sulfide gas sensing toward on-site monitoring of chilled meat spoilage based on ratio-type fluorescent probe. Food Chem..

[9] Li H., Tang R., Mustapha W.A., Liu J., Hasan K.M., Li X., Huang M. (2021). Application of Gelatin Composite Coating in Pork Quality Preservation during Storage and Mechanism of Gelatin Composite Coating on Pork Flavor. Gels.

[10] Jia W., Li R., Wu X., Liu L., Liu S., Shi L. (2021). Molecular mechanism of lipid transformation in cold chain storage of Tan sheep. Food Chem..

[11] Karabagias I.K. (2018). Volatile Profile of Raw Lamb Meat Stored at 4 ± 1 °C: The Potential of Specific Aldehyde Ratios as Indicators of Lamb Meat Quality. Foods.

[12] Xiang C., Li S., Liu H., Liang C., Fang F., Zhang D., Wang Z. (2021). Impact of Chilling Rate on the Evolution of Volatile and Non-Volatile Compounds in Raw Lamb Meat during Refrigeration. Foods.

[13] Zhao B., Sun B., Wang S., Zhang Y., Zang M., Le W., Wang H., Wu Q. (2021). Effect of different cooking water on flavor characteristics of mutton soup. Food Sci. Nutr..

[14] Wang Y., Luo R., Wang S. (2022). Study on key aroma compounds in the electric roasting process of Tan mutton. J. Food Process. Preserv..

[15] Liu C., Chu Z., Weng S., Zhu G., Han K., Zhang Z., Huang L., Zhu Z., Zheng S. (2022). Fusion of electronic nose and hyperspectral imaging for mutton freshness detection using input-modified convolution neural network. Food Chem..

[16] Chen J., Yan W., Fu Y., Wang L., Lv X., Dai R., Li X., Jia F. (2022). The Use of Electronic Nose in the Quality Evaluation and Adulteration Identification of Beijing-You Chicken. Foods.

[17] Wang Q., Li L., Ding W., Zhang D., Wang J., Reed K., Zhang B. (2019). Adulterant identification in mutton by electronic nose and gas chromatography-mass spectrometer. Food Control.

[18] Lucy H., Rebecca C., Damiana S.N., Rachael S. (2022). Volatile and semi-volatile compounds in flavoured hard seltzer beverages: Comparison of high-capacity sorptive extraction (HiSorb) methods. Adv. Sample Prep..

[19] Soteria E., Marinos S., Agapios A. (2022). Aroma characterization of raw and electrochemically treated goat whey wastewater. Sustain. Chem. Pharm..

[20] Cheng Z., Mannion D.T., O’Sullivan M.G., Miao S., Kerry J.P., Kilcawley K.N. (2021). Comparison of Automated Extraction Techniques for Volatile Analysis of Whole Milk Powder. Foods.

[21] Gallego E., Perales J.F., Calaf J.M. (2023). Continuous monitoring of volatile organic compounds through sensorization. Automatic sampling during pollution/odour/nuisance episodic events. Atmos. Environ..

[22] Elorduy I., Durana N., Antonio Garcia J., Carmen Gomez M., Alonso L. (2018). Evaluation of Uncertainty Associated with Determination of Particle-bound PAHs in Ambient Area by TD-GC/MS and Soxhlet-GC/MS. Aerosol Air Qual. Res..

[23] Kim Y.-M., Kim J.W., Moon H.M., Lee M.-J., Hosaka A., Watanabe A., Teramae N., Park Y.-K., Myung S.-W. (2017). Rapid Quantification of N-Methyl-2-pyrrolidone in Polymer Matrices by Thermal Desorption-GC/MS;Original Papers. Anal. Sci..

[24] Liu G., Fang S., Wang Y., Liu J., Liang Y., Cao T., Liu Q. (2023). Emission of Volatile Organic Compounds in Crumb Rubber Modified Bitumen and Its Inhibition by Using Montmorillonite Nanoclay. Polymers.

[25] Ji C., You L., Luo R. (2022). Proteomics and metabolomics combined study on endopathic changes of water-soluble precursors in Tan lamb during postmortem aging. Food Sci. Nutr..

[26] Wang L., Liu T., Liu L., Liu Y., Wu X. (2022). Impacts of chitosan nanoemulsions with thymol or thyme essential oil on volatile compounds and microbial diversity of refrigerated pork meat. Meat Sci..

[27] Chen Q., Hu Y., Wen R., Wang Y., Qin L., Kong B. (2020). Characterisation of the flavour profile of dry fermented sausages with different NaCl substitutes using HS-SPME-GC-MS combined with electronic nose and electronic tongue. Meat Sci..

[28] North M.K., Zotte A.D., Hoffman L.C. (2019). The effects of dietary quercetin supplementation on the meat quality and volatile profile of rabbit meat during chilled storage. Meat Sci..

[29] Kilgannon A.K., Holman B.W.B., Frank D.C., Mawson A.J., Collins D., Hopkins D.L. (2020). Temperature-time combination effects on aged beef volatile profiles and their relationship to sensory attributes. Meat Sci..

[30] Watkins P.J., Jaborek J.R., Teng F., Day L., Castada H.Z., Baringer S., Wick M. (2021). Branched chain fatty acids in the flavour of sheep and goat milk and meat: A review. Small Rumin. Res..

[31] Ojeda-Amador R.M., Fregapane G., Salvador M.D. (2020). Influence of cultivar and technological conditions on the volatile profile of virgin pistachio oils. Food Chem..

[32] Wen R., Kong B., Yin X., Zhang H., Chen Q. (2021). Characterisation of flavour profile of beef jerky inoculated with different autochthonous lactic acid bacteria using electronic nose and gas chromatography–ion mobility spectrometry. Meat Sci..

[33] Bai S., Wang Y., Luo R., Ding D., Bai H., Shen F. (2020). Characterization of flavor volatile compounds in industrial stir-frying mutton sao zi by GC-MS, E-nose, and physicochemical analysis. Food Sci. Nutr..

[34] Wang B., Wang Z., Chen Y., Liu X., Liu K., Zhang Y., Luo H. (2021). Carcass Traits, Meat Quality, and Volatile Compounds of Lamb Meat from Different Restricted Grazing Time and Indoor Supplementary Feeding Systems. Foods.

[35] Han G., Zhang L., Li Q., Wang Y., Chen Q., Kong B. (2019). Impacts of different altitudes and natural drying times on lipolysis, lipid oxidation and flavour profile of traditional Tibetan yak jerky. Meat Sci..

[36] Wang D., Zhang J., Zhu Z., Lei Y., Huang S., Huang M. (2022). Effect of ageing time on the flavour compounds in Nanjing water-boiled salted duck detected by HS-GC-IMS. LWT.

[37] Ma X., Yang D., Qiu W., Mei J., Xie J. (2021). Influence of Multifrequency Ultrasound-Assisted Freezing on the Flavour Attributes and Myofibrillar Protein Characteristics of Cultured Large Yellow Croaker (*Larimichthys crocea*). Front. Nutr..

[38] Zhang Z., Wu R., Gui M., Jiang Z., Li P. (2021). Identification of the Specific Spoilage Organism in Farmed Sturgeon (*Acipenser baerii*) Fillets and Its Associated Quality and Flavour Change during Ice Storage. Foods.

[39] Insausti K., Murillo-Arbizu M.T., Urrutia O., Mendizabal J.A., Beriain M.J., Colle M.J., Bass P.D., Arana A. (2021). Volatile Compounds, Odour and Flavour Attributes of Lamb Meat from the Navarra Breed as Affected by Ageing. Foods.

[40] Larick D.K., Turner B.E. (1990). Headspace Volatiles and Sensory Characteristics of Ground Beef from Forage- and Grain-Fed Heifers. J. Food Sci..

[41] Zhu X., Li Q., Li J., Luo J., Chen W., Li X. (2018). Comparative Study of Volatile Compounds in the Fruit of Two Banana Cultivars at Different Ripening Stages. Molecules.

[42] Muhammad S., Muhammad A.F., Sajid A.M., Muhammad I., Ali I., Shahzad H. (2017). Oxidative stability and lipid oxidation flavoring volatiles in antioxidants treated chicken meat patties during storage. Lipids Health Dis..

[43] Chen L., Zeng W., Rong Y., Lou B. (2021). Characterisation of taste-active compositions, umami attributes and aroma compounds in Chinese shrimp. Int. J. Food Sci. Technol..

[44] Su D., He J.J., Zhou Y.Z., Li Y.L., Zhou H.J. (2022). Aroma effects of key volatile compounds in Keemun black tea at different grades: HS-SPME-GC-MS, sensory evaluation, and chemometrics. Food Chem..

[45] Wang J., Chen L., Liu Y., Olajide T.M., Jiang Y., Cao W. (2022). Identification of key aroma-active compounds in beef tallow varieties using flash GC electronic nose and GC × GC-TOF/MS. Eur. Food Res. Technol..

[46] Liu H., Hui T., Fang F., Ma Q., Li S., Zhang D., Wang Z. (2021). Characterization and Discrimination of Key Aroma Compounds in Pre- and Postrigor Roasted Mutton by GC-O-MS, GC E-Nose and Aroma Recombination Experiments. Foods.

[47] Beldarrain L.R., Morán L., Sentandreu M.Á., Barron L.J., Aldai N. (2022). Effect of ageing time on the volatile compounds from cooked horse meat. Meat Sci..

[48] Zhang D., Ji W., Peng Y., Ji H., Gao J. (2020). Evaluation of Flavor Improvement in Antarctic Krill Defluoridated Hydrolysate by Maillard Reaction Using Sensory Analysis, E-nose, and GC-MS. J. Aquat. Food Prod. Technol..

[49] Duan S., Tang X., Li W., Huang X. (2023). Analysis of the Differences in Volatile Organic Compounds in Different Muscles of Pork by GC-IMS. Molecules.

[50] Dan T., Hu H., Li T., Dai A., He B., Wang Y. (2022). Screening of mixed-species starter cultures for increasing flavour during fermentation of milk. Int. Dairy J..

[51] Giannetti V., Mariani M.B., Torrelli P., Marini F. (2019). Flavour component analysis by HS-SPME/GC–MS and chemometric modeling to characterize Pilsner-style Lager craft beers. Microchem. J..

[52] Munekata P.E., Finardi S., de Souza C.K., Meinert C., Pateiro M., Hoffmann T.G., Domínguez R., Bertoli S.L., Kumar M., Lorenzo J.M. (2023). Applications of Electronic Nose, Electronic Eye and Electronic Tongue in Quality, Safety and Shelf Life of Meat and Meat Products: A Review. Sensors.

[53] Zhao L., Zhang H., Huang F., Liu H., Wang T., Zhang C. (2023). Authenticating Tibetan pork in China by tracing the species and geographical features based on stable isotopic and multi-elemental fingerprints. Food Control.

[54] Qiu H., Qu K., Eun J.B., Zhang H. (2023). Analysis of thermal oxidation of different multi-element oleogels based on carnauba wax, β-sitosterol/lecithin, and ethyl cellulose by classical oxidation determination method combined with the electronic nose. Food Chem..

[55] Liu J., Hu Z., Liu D., Zheng A., Ma Q. (2023). Glutathione metabolism-mediated ferroptosis reduces water-holding capacity in beef during cold storage. Food Chem..

